# Biodegradation of Allethrin by a Novel Fungus *Fusarium proliferatum* Strain CF2, Isolated from Contaminated Soils

**DOI:** 10.3390/microorganisms8040593

**Published:** 2020-04-20

**Authors:** Pankaj Bhatt, Wenping Zhang, Ziqiu Lin, Shimei Pang, Yaohua Huang, Shaohua Chen

**Affiliations:** 1 State Key Laboratory for Conservation and Utilization of Subtropical Agro-bioresources, Guangdong Province Key Laboratory of Microbial Signals and Disease Control, Integrative Microbiology Research Centre, South China Agricultural University, Guangzhou 510642, China; pankajbhatt.bhatt472@gmail.com (P.B.); 20191047008@stu.scau.edu.cn (W.Z.); 20192047010@stu.scau.edu.cn (Z.L.); 20192047012@stu.scau.edu.cn (S.P.); 20183138021@stu.scau.edu.cn (Y.H.); 2 Guangdong Laboratory for Lingnan Modern Agriculture, Guangzhou 510642, China

**Keywords:** allethrin, *Fusarium proliferatum*, biodegradation, metabolic pathway, kinetics

## Abstract

Continuous use of allethrin has resulted in heavy environmental contamination and has raised public concern about its impact on human health, yet little is known about the kinetics and microbial degradation of this pesticide. This study reported the degradation kinetics in a novel fungal strain, *Fusarium proliferatum* CF2, isolated from contaminated agricultural fields. Strain CF2 utilized 50 mg·L^−1^ of allethrin as the sole carbon source for growth in minimal salt medium and tolerated high concentrations of allethrin of up to 1000 mg·L^−1^. The optimum degradation conditions for strain CF2 were determined to be a temperature of 26 °C and pH 6.0 using response surface methodology. Under optimum conditions, strain CF2 completely degraded allethrin within 144 hours. The degradation kinetics of allethrin followed first order reaction kinetics. Kinetics analysis showed that its half-life was substantially reduced by 507.1 hours, as compared to the uninoculated control. This study provides new insights into the microbial degradation of allethrin with fungal *F. proliferatum* CF2.

## 1. Introduction

Allethrin (2-methyl-4-oxo-3-(2-propanyl)-2-cyclopenten-lyl-2-2-dimethyl-3-(2-methyl-1-propenyl cyclopropane)-carboxylate) is a synthetic pyrethroid compound that is widely used to control flies and mosquitoes in the home, crawling and flying insects on animal farms, and fleas and ticks on dogs and cats [1]. Allethrin was the first synthetic pyrethroid synthesized in the USA by Milton S. Schechter in 1949. Like all pyrethroids, it acts as an axonic poison that binds and stops the function of sodium ion channels, resulting in hyperactivity of the nervous system and, finally, paralysis and death of insects [2]. Allethrin has a broad application in mosquito-borne diseases and agricultural pest control [3]. However, the widespread application of allethrin causes heavy environmental contamination and also affects human health [4]. The large-scale use of domestic pesticides as mosquito repellents is greatly increasing concerns about its toxicity. Applied pesticides reach natural targets such as air, water, and soil. Pyrethroids constitute about 25% of the total global insecticide market, indicating its importance and application value [5]. On the basis of their chemical structure and mechanism, pyrethroids are divided into two broad categories, namely, Type I and Type II. Allethrin is a Type I pyrethroid because it lacks the *α*-cyano group [6,7].

Allethrin is released from different households and agricultural operations and persists in the environment for a long time. Mammals’ degradation of allethrin leads to the formation of free radicals that cause DNA damage and damage to the integrity of the membrane, which causes cell death [8,9]. Allethrin-induced reactive species create oxidative cell damage in human erythrocyte cells [10]. Neurotoxicity in the presence of allethrin has been reported in rodents [10]; it causes genotoxicity in human peripheral blood lymphocytes, as well as in Swiss albino mice [11,12]. In humans, allethrin causes toxicity in corneal epithelial cells through mitochondrial-mediated apoptosis [13], and the biochemical profiles of plasma cells in humans are altered by allethrin [14].

Due to the large-scale application of pyrethroids in the agricultural system, they can persist for a long time on the basis of biotic and abiotic factors [15]. These chemicals reach the bottom layers of the soil and thus might contaminate groundwater bodies. Finally, the concentration increases in the ecosystem via various direct and indirect routes through the biomagnification process [16]. Biodegradation is an eco-friendly approach to remove pyrethroid residues from the environment [17,18,19], and bacterial-based biodegradation of pesticides is emphasized more compared to fungal strains [20]. However, several reports have identified the potential fungal genera for pyrethroid degradation. These microbial strains have the potential to utilize pyrethroids as a sole source of carbon and nitrogen, such as *Aspergillus*, *Candida*, *Trichoderma*, *Eurotium*, and *Cunninghamella* [21,22,23,24,25,26,27,28]. Fungi may produce other intracellular and extracellular enzymes that play an important role in pyrethroid degradation. For the biodegradation of pesticides, fungi are considered to be the better potential candidates compared to bacteria [20]. Naturally, white rot fungi have the robustness for degradation of recalcitrant chemicals in the environment [29,30]. Pyrethroids were firstly degraded by cleavage of the ester bond, due to the presence of an enzyme known as pyrethroid hydrolase [31,32]. Among pyrethroids, allethrin has been given less emphasis in degradation research—only the allethrin-degrading bacteria *Acidomonas* sp. and *Sphingomonas trueperi* have been identified previously [2,33,34].

To date, little is known about allethrin biodegradation by fungi associated with metabolic behaviors and kinetics. Therefore, the aim of the study was: (1) to isolate and identify allethrin-degrading fungi from contaminated agricultural soils; (2) to investigate the degradation kinetics of allethrin in minimal salt medium; (3) to optimize the degradation conditions with isolated fungi; and (4) to characterize the metabolic pathway of allethrin. The results of this study will help to explore the role of fungi in the degradation of allethrin and similar pesticides.

## 2. Materials and Methods

### 2.1. Chemicals and Media

Technical grade allethrin and other synthetic pyrethroids used for the present experiment were purchased from Sigma-Aldrich, St. Louis, MO, USA. The acetonitrile used for the chromatographic analysis was obtained from Sigma-Aldrich, USA. Allethrin was dissolved in acetone at a stock concentration of 10 g·L^−1^ and stored in a dark bottle in a refrigerator at 4 °C. This stock was used for the degradation analysis of allethrin. The mineral salt medium (MSM) containing 2 g (NH_4_)_2_SO_4_, 0.2 g MgSO_4_·7H_2_O, 0.01 g CaCl_2_·2H_2_O, 0.001 g FeSO_4_·7H_2_O, 1.5 g Na_2_HPO_4_·12H_2_O, and 1.5 g KH_2_PO_4_ per liter was used for the biodegradation assays in the study. Czapek–Dox medium (CDM) containing (in grams per liter) sucrose, 30; NaNO_3_, 2; KCl, 0.5; MgSO_4_, 0.5; K_2_HPO_4_, 1; Fe_2_(SO_4_)_3_, 0.01; and peptone, 0.5, was used for the cultivation of fungal strains in the laboratory. Both media were autoclaved at 121 °C for 20 min before fungal strain inoculation.

### 2.2. Enrichment and Isolation of Allethrin-Degrading Fungi

Soil samples were collected from the contaminated agricultural fields of the South China Agricultural University, Guangzhou, China and used to isolate fungi that degrade allethrin. Collected soil samples were stored at 4 °C for further use. Enrichment and isolation of the allethrin-degrading strain was carried out in MSM using enrichment culture techniques [33,34,35,36,37]. The fungal colonies of different morphologies were collected and purified with the streaking method. The isolated strains were further screened for their abilities to degrade allethrin. The pure fungal isolate with the highest allethrin degradation ability was designated as CF2 for further study.

### 2.3. Identification of the Strain CF2

The fungal isolate CF2 was grown in CDM agar media and investigated by a light microscope. The colonies were observed with the microscope on different days. Total genomic DNA of the strain CF2 was isolated using an OMEGA fungal DNA extraction mini kit (Omega Bio-Tek, Norcross, GA, USA). The 5.8 S rDNA gene was amplified using polymerase chain reaction (PCR) with the universal primer sets ITS-5 (5’-GGAAGTAAAAGTCGTAACAAGG-3’) and ITS-4 (5’-GCATATCAATAAGCGGAGGA-3’) [17]. The conditions for PCR was as follows—initial denaturation at 95 °C up to 3 min, followed by 35 cycles of reactions with denaturation at 94 °C for 1 min, annealing at 55 °C for 1 min, and extension at 72 °C for 1 min. The last cycle was followed by 10 min extension at 72 °C. The PCR products were observed in 1% agarose gel electrophoresis with a 2 Kb marker and sent for sequencing. The resulting sequences were compared with the GenBank submitted sequence database using the National Centre for Biotechnology Information (NCBI) BLAST search. Multiple sequence alignment was performed among sequences using the Clustal-W offline tool. Phylogenetic analysis was performed using the MEGA-X software (Pennsylvania State University, University Park, PA, USA). The phylogenetic tree of all of the sequences was formed using unweighted pair group method with arithmetic mean (UPGMA) techniques.

### 2.4. Preparation of Fungal Inoculum

The fungal strain CF2 was grown in 50 mL of CDM for two days at 28 °C, with a shaking speed of 200 rpm. The mycelia of strain CF2 were harvested using centrifugation at 5000 rpm for 10 min and washed with 0.9% sterile saline [38]. The washed mycelia were resuspended in 50 mL saline; 5 mL of this suspension was used as inoculum for allethrin degradation.

### 2.5. Optimization of the Degradation Conditions for Strain CF2

To study the effects of temperature and pH on the growth/degradation of allethrin by strain CF2, a single-factor experiment was designed under different temperatures (18, 22, 26, 30, 34 °C) and pH (4.0, 5.0, 6.0, 7.0, 8.0) conditions. Strain CF2 was incubated in MSM (pH 6.0) containing 50 mg·L^−1^ allethrin at 26 °C at 110 rpm on a rotary shaker for 5 days. Each treatment was performed in triplicate with noninoculated samples as a control [21]. The growth of strain CF2 was determined by measurement of the dry weight of the mycelium. The residual concentration of allethrin was analyzed by high-performance liquid chromatography (HPLC) (Waters e2695, Milford, MA, USA).

The culture conditions for allethrin degradation were further optimized by response surface methodology (RSM) with strain CF2. The pH, temperature, and time of incubation were considered as significant for allethrin degradation, based on the results of previous single-factor experiments [39,40]. Central composite design (CCD) of five levels (−1.68, −1, 0, +1, +1.68) was used for optimization with 24 experiments with three replicates of Design Expert-11 software. The used variables were coded according to the following Equation (1):

*x*_i_ = (*X*_i_ − *X*_0_)/Δ*X*_i_ (1)

where *x*_i_ is the dimensionless value of an independent variable; *X*_i_ is the real value of an independent variable; *X*_0_ is the real value of an independent variable at the center point; and Δ*X*_i_ is the step change of the real value of a variable.

Allethrin (50 mg·L^−1^) degradation in 50 mL MSM was considered as the dependent variable after 5 days. Data were analyzed by a regression procedure of RSM to fit the quadratic polynomial Equation (2).

*Y*_i_ = *b*_0_ + ∑ *b*_i_*X*_i_ + ∑ *b*_ij_*X*_i_*X*_j_ + ∑ *b*_ii_*X*^2^_i_ (2)

where *Y*_i_ is predicted response, *X*_i_ and *X*_j_ are variables, *b*_0_ is constant, *b*_i_ is the linear coefficient, *b*_ij_ is the interaction coefficient and *b*_ii_ is the quadratic coefficient.

### 2.6. Degradation Kinetics of Allethrin with Strain CF2

To check the degradation kinetics of allethrin, strain CF2 was inoculated in the MSM under the influence of optimal culture conditions. The final concentration of 50 mL MSM in a 250 mL Erlenmeyer flask was 50 mg∙L^−1^. The flasks were incubated in triplicate with a control at 26 °C for up to 144 h. The samples were collected at 24 h intervals and the residual concentration was determined by HPLC. The growth of strain CF2 was determined by measurement of the dry weight of the mycelium. Each treatment was set in triplicate with noninoculated samples as a control [41].

### 2.7. Extraction and HPLC Analysis

The allethrin and its intermediate metabolites after degradation were extracted using acetone and ethyl acetate methods. Ten milliliters of the MSM after different days of the experiment was collected in 50 mL tubes. Furthermore, 10 mL of acetone was added in each of the tubes, followed by ultrasonication for 15 min. The 10 mL of ethyl acetate was added in the sonicated samples and vortexed well. The separated layer of the organic and aqueous phase was clearly distinguished, and the organic phase was collected in new tubes. Again, the 10 mL of ethyl acetate was added for complete recovery of the allethrin from the samples. Then, 10 g of anhydrous sodium sulfate was added to each of the tubes and vortexed. All of the tubes were centrifuged at 8000 rpm for 4 min. Then, the samples were evaporated under a vacuum in round-bottom flasks, and the recovery of allethrin and its metabolites were used for HPLC analysis [23,42,43,44,45].

HPLC was used for the quantitative analysis of allethrin residue in each experiment throughout the work. HPLC was attached with a UV detector and a C_18_ reversed column, and a column oven was used for the detection and quantification of allethrin. The mobile phase was composed of deionized water and acetonitrilem, which was filtered before use, using a 0.45 µm filter under vacuum pressure. The prepared mobile phase was set in acetonitrile (65)/water (35) with a flow rate of 0.7 mL·min^−1^ and the pump mode was isocratic. The volume used for sample injection was 20 µL. UV detection was carried out at 250 nm, with a 34 min running time.

### 2.8. Kinetic Analysis

The allethrin degradation parameters were calculated as per first-order reaction kinetics [21,46,47,48].

(3) $$C_{t}=C_{0}e^{-kt}$$

where *C*_0_ is the initial concentration of allethrin in MSM, *C_t_* is the concentration of allethrin at time *t*, *k* is the degradation rate constant (h^−1^), and *t* is the reaction time (h).

The degradation half-life (*t*_1/2_) of allethrin was determined as Equation (4).

(4) $$t_{1/2}=\ln2/k$$

### 2.9. Statistical Analysis

Statistical data regarding allethrin degradation in all of the experimental sets were subjected to two-way analysis of variance (ANOVA) to calculate the effects of the treatments on allethrin degradation. Allethrin concentration and fungi treatments were considered as first and second factors, respectively. The critical difference (CD), calculated at the 5% level of significance, was used to compare the differences among treatment means. The statistical analysis of allethrin biodegradation was carried out in IBM-SPSS version 20.0, USA.

## 3. Results

### 3.1. Isolation and Identification of Strain CF2

**Figure 1:**
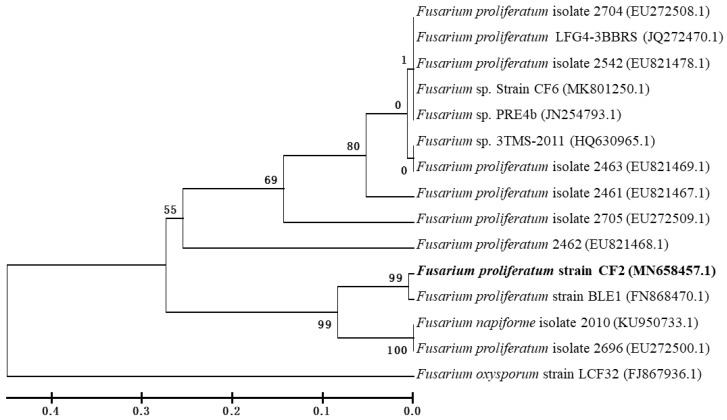
Phylogenetic relationships of the isolated strain CF2 and other closely related species on the basis of 5.8S rRNA sequences. The numbers at the nodes represent the accession numbers of GenBank. The bootstrap value for the unweighted pair group method with arithmetic mean (UPGMA) phylogenetic tree was 1000.

After four rounds of enrichment at different allethrin concentrations (50–1000 mg·L^−1^), six fungal isolates were recovered from the soil samples. The fungal isolate designated as CF2 was selected and screened on the basis of its potential allethrin degradation abilities for further experiment. The fungus efficiently degraded and metabolized allethrin up to concentrations as high as 1000 mg·L^−1^ in MSM. Strain CF2 is an obligatory aerobic fungus with spore-forming potential. Its colonies are white, with dense mycelia and rounds covering the entire CDM plates (Supplementary Figures S1 and S2). Phylogenetic analysis of 5.8 S rDNA gene confirmed that strain CF2 has a high similarity to *Fusarium proliferatum* (Figure 1). The GenBank accession number of the submitted strain CF2 is MN 658457.1. Based on morphology and molecular tools, strain CF2 was identified as *F. proliferatum*.

### 3.2. Optimization of Allethrin Degradation Conditions

**Figure 2:**
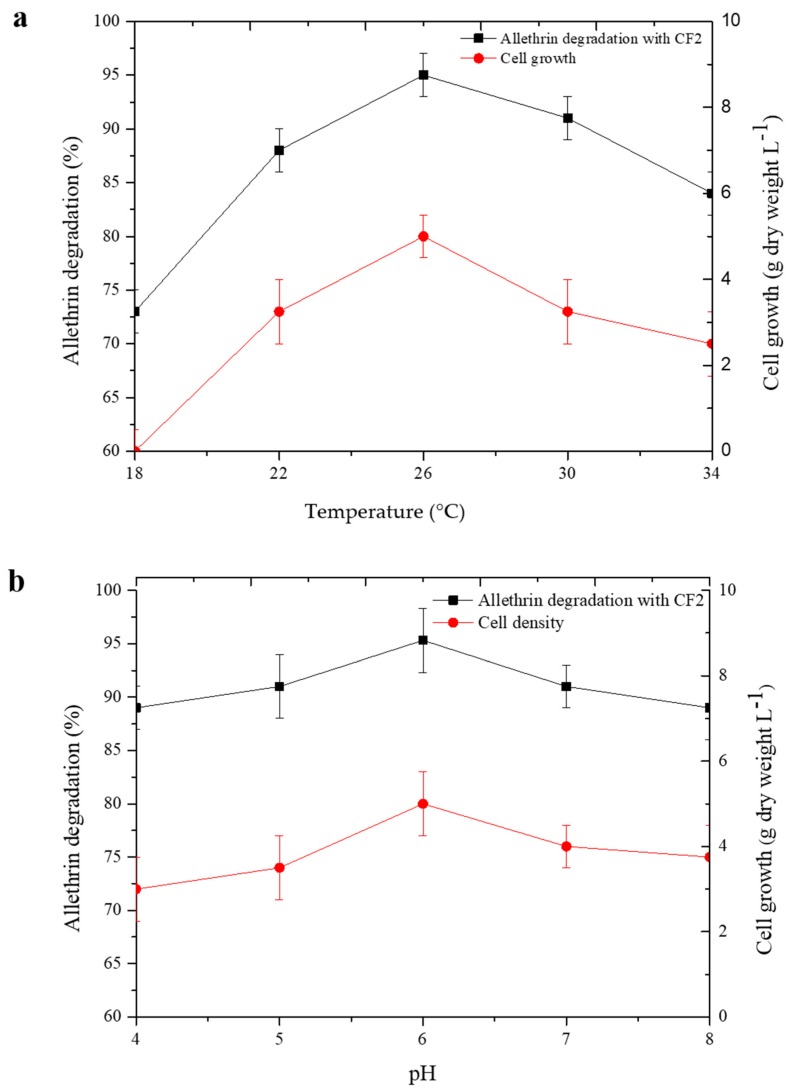
Effect of temperature (**a**) and pH (**b**) on the degradation of allethrin by strain CF2. The data presented are mean ± standard errors of three independent experiments.

Strain CF2 effectively degraded allethrin under different temperatures and pH conditions (Figure 2). It degraded 73.1%, 87.9%, 95.0%, 91.3%, and 84.2% of allethrin at 18, 22, 26, 30, and 34 °C within 5 days, respectively. A total of 89.2%, 91.0%, 95.3%, 91.7%, and 89.4% degradation was achieved at pH 4.0, 5.0, 6.0, 7.0, and 8.0, respectively. Maximum degradation and mycelium dry weight were both observed at a temperature of 26 °C and pH 6.0.

Central composite design (CCD) of response surface methodology (RSM) was further used to investigate the effects of each of the parameters on allethrin degradation (Supplementary Figure S3). The interactive effects of the pH (A), temperature (B), and culture time (C) on allethrin degradation were determined with strain CF2. The matrix of the design used with the optimized responses is given in Table 1. The data of 24 experiments were used for analysis with Design Expert-11. The results of quadratic polynomial model fitting in terms of analysis of variance (ANOVA) are shown in Supplementary Table S1. The *R*^2^ value 0.9823, showing the predicted value of model, is consistent with the experimental values. The *F*-value of 143.23 and a *p*-value < 0.05 indicates that the model parameters are statistically significant. Regression-based analysis of the experimental data formulated the following coded quadratic polynomial equation explaining allethrin degradation.

Y = 94.23 − 0.1231 × A − 0.2929 × B + 4.04 × C − 1.25 × AB + 2.00 × AC − 4.50 × BC − 6.74 × A^2^ − 6.91 × B^2^ − 5.11 × C^2^

where Y = % allethrin degradation using the strain CF2; and A, B, and C are the coded terms for pH, temperature (°C), and incubation time (days), respectively. The statistical significance of the data was evaluated using *F* test and *t*-test. All of the statistical analyses suggest that the model linear term coefficients of A, B, and C showed significant effects (*p* < 0.05) on allethrin degradation. The coefficient estimates represent the expected change in response per unit change in factor value when all remaining factors were held constant. The predicted *R*^2^ value 0.9412 supports the applied model. The results show that there is a good correlation between the predicted value of the model and the experimental value. The low coefficient of variation (CV = 1.64) demonstrates the good precision and reliability of the performed experiment. Thus, the developed model could be adequate for the determination of a range of variables.

**Figure 3:**
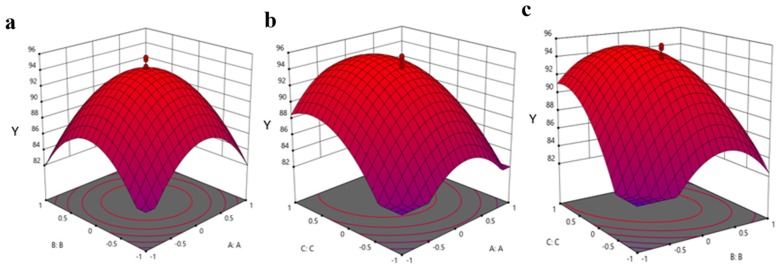
Optimization of the allethrin degradation condition using response surface methodology. Response surface plots showing the interactive effects of pH (**a**), temperature (**b**), and incubation time (**c**) on allethrin degradation (Y) with strain CF2.

**Figure 4:**
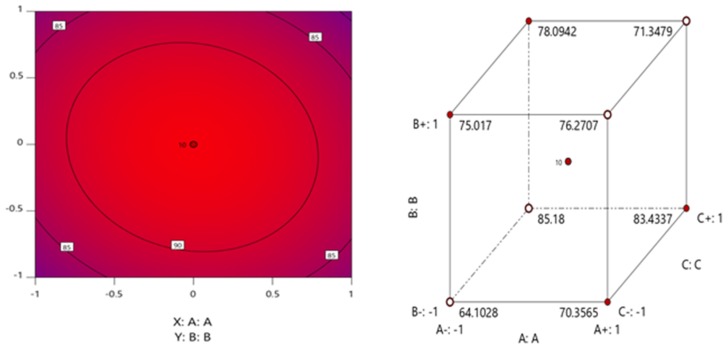
Contour plot representing the center-optimized point for allethrin degradation, and the cube structure representing the effects of pH (**A**), temperature (**B**), and incubation time (**C**) on allethrin degradation.

**Figure 5:**
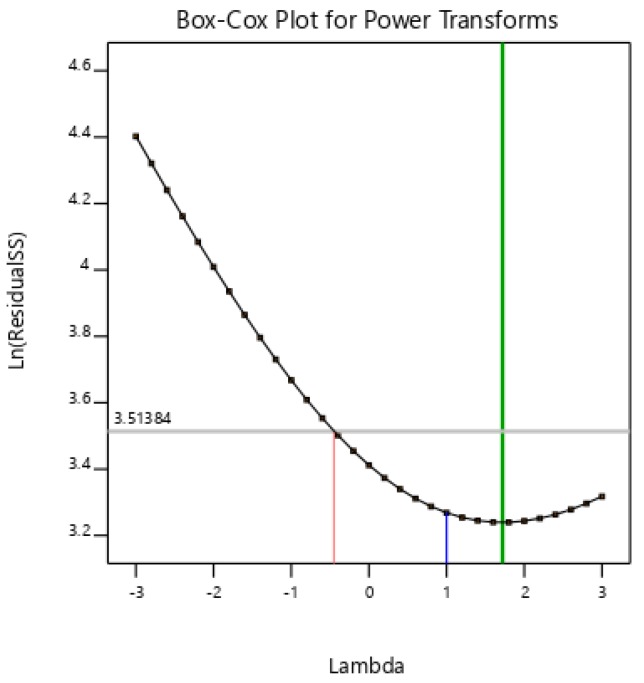
A Box–Cox plot representing the correlation coefficients for various optimized conditions. The green line shows the best lambda at value 1.72. The class interval for lambda (–0.45, 4.24) are shown. Values are mean ± SEM of triplicates and are significant at *p* < 0.05 for all treatments.

The three-dimensional response surface plots of all of the variables determined the effect of each parameter with interactive points for allethrin degradation with strain CF2 (Figure 3). This model predicted a maximum allethrin degradation of 95.6% at the stationary point. The optimum points for degradation in the model for A, B, and C were 0, 0, and 0 in terms of coded units corresponding to pH 6.0, temperature 26 °C, and an incubation time of 5 days, respectively. The contour plot and cube represent graphs showing the interactive effect of each of the parameters in allethrin degradation with strain CF2 (Figure 4). The center point in the contour plot with run 10 represents the maximum allethrin degradation. The detailed analysis each of the 24 runs with the actual value, predicted value, residuals, leverage, and cooks distance supports the degradation of allethrin being highly significant with strain CF2. The cook’s distance for the treatments was in the range of 0.01–0.048. The statistical values calculated for the model suggest that the model is effective for allethrin degradation with fungal strain CF2. The residuals vs. run and the predicted analysis represent the lurking variables that influence allethrin degradation using strain CF2. The externally studentized residuals were calculated from −3.8 to +3.8 (*p* < 0.05) for all of the treatments with strain CF2 during allethrin degradation. Furthermore, Box–Cox analysis shows that the used model justifies the best degradation model for allethrin degradation (Figure 5).

### 3.3. Degradation Kinetics of Allethrin with Strain CF2

**Figure 6:**
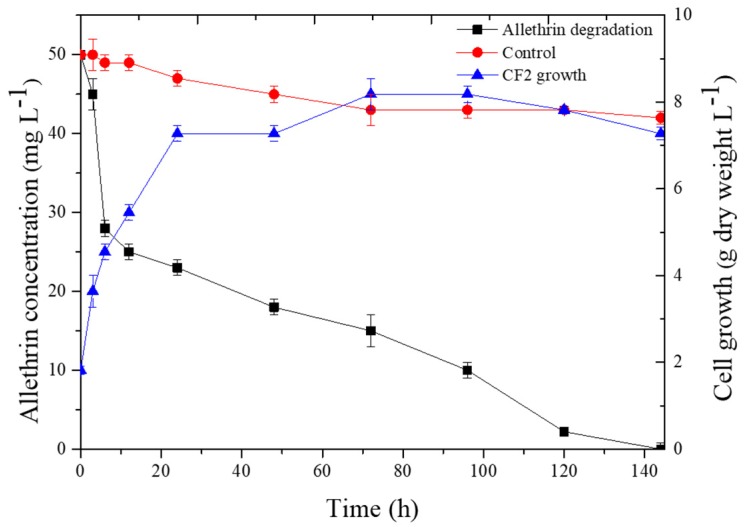
Growth curves of strain CF2 and allethrin degradation profiles over time. The data presented are mean ± standard errors of three independent experiments.

Kinetics-based analysis of allethrin degradation was observed with different time intervals. The degradation kinetics of allethrin with strain CF2 are shown in Figure 6. Strain CF2 rapidly degraded allethrin after inoculation in the MSM. We observed that 95.6% of allethrin (50 mg·L^−1^) was degraded within 120 h of the experiment without a lag phase. After 144 h, no allethrin residue was found in the experimental group. However, the dissipation of noninoculated control was only 18.0%. Microbial degradation was associated with mycelial growth. Mycelial growth was highest during the first 24 h of incubation and a maximum mycelium dry weight was observed after 72 h.

The kinetic parameters were calculated with strain CF2. The degradation rate constant (*k*) and half-time (*t*_1/2_) for the control were calculated as 0.0013 h^−1^ and 533.19 h, respectively, whereas for CF2-treated samples, these values were 0.0193 h^−1^ and 26.05 h, respectively. The determination coefficient (*R*^2^) was 0.926 and 0.850 for the control and CF2 treatment group, respectively. These results confirm that inoculation with strain CF2 significantly accelerated allethrin degradation in MSM. At the end of the experiment, allethrin was completely degraded by the *F. proliferatum* strain CF2.

## 4. Discussion

Due to the large-scale use of allethrin at various agricultural locations and in the home, residual concentrations are increasing day-by-day and are causing hazardous effects on living cells in the environment. Bioremediation was previously found to be the most promising approach for the removal of pesticide residues from the environment [49,50,51,52,53,54]. In the present study, a novel fungal strain CF2, which was identified as *F. proliferatum*, responsible for degradation of allethrin, was isolated from pesticide-contaminated agricultural fields. To date, the degradation assay information about allethrin is very limited. No one has characterized the fungi-based degradation of allethrin, and to the best of our knowledge, this is the first report on this topic.

Scant information is available for the degradation of pyrethroids and other pesticides using fungi. Previously, *Trichoderma*, *Aspergillus*, *Eurotium*, *Candida*, and *Phanerochaete* have been reported to degrade pyrethroids [22,23,24,25,55]. *Eurotium cristatum* ET1, a fungus from fu brick tea in China, was found to degrade β-cypermethrin and 3-phenoxybenzaldehye [55]. Pyrethroid-degrading fungi *Cunninghamella elegans* was characterized for the degradation of cyhalothrin [25]. Fungi have the great potential to degrade xenobiotics from the environment by chemical modification and by influencing the bioavailability [56]. However, there is still little attention given to fungi in pesticide degradation as compared to bacteria. On the basis of biomass fungi dominating the kingdom in soil and the aqueous system, they deserve special attention for pesticide degradation [27,57]. Naturally, fungi are found to be more advantageous than bacteria for the degradation of recalcitrant compounds [20,58]. To identify effective fungi for pesticide degradation, our group identified *Cladosporium* and *Candida* for the degradation of pesticides [22,27,35]. However, the potential use of *F. proliferatum* in biodegradation of pesticides has not received the attention it deserves. Our studies, focused on *F. proliferatum*, showed that it could utilize allethrin as the sole source of carbon for growth, and thus it can be successfully colonized in nutrient-deficient niches.

We observed that the *F. proliferatum* strain CF2 has the ability to degrade allethrin at a wide variety of pH levels, temperatures, and incubation times. These characteristics of strain CF2 may be beneficial for the degradation at various naturally contaminated sites due to its survival in different conditions. Previous studies have confirmed that fungal strains that can perform good degradation in adverse conditions could also be beneficial for in situ remediation in indigenous environments [27,35]. Under high acidic environments, strain CF2 did not perform well in degradation. These results are consistent with previous findings that reported that rapid degradation was liable to happen under neutral- and alkaline-trending conditions [36,59,60]. Indeed, our results imply that the synthesis and expression of the degradation enzyme of strain CF2 may perform better under neutral- and alkaline-trending conditions. The strain CF2 participated in efficient degradation of allethrin in adverse environments, which suggests that it may be used as a suitable candidate for the bioremediation of allethrin and other pyrethroids.

RSM was used for optimization of the degradation condition with strain CF2. RSM is an analytical tool for the optimization of various biological processes and has been used previously with bacterial and fungal process optimization [18,22,36,39]. Previous studies support the use of RSM in pesticide degradation as being useful for the optimization of processes with precision [61,62,63,64]. In our study, we conducted a detailed analysis of allethrin degradation with a central composite design of RSM. The results showed that the optimum degradation conditions for strain CF2 were pH 6.0, temperature 26 °C, and an incubation time of 5 days. Under these conditions, strain CF2 degraded allethrin (50 mg∙L^−1^) by up to 95.6% within 5 days. The contour plot, cube, residual vs. predicted, and Box–Cox plot etc. analyses confirmed that the used model is efficient for allethrin degradation optimization. Similar results were previously observed on Cladosporium sp. HU for the degradation of fenvalerate [22].

It is noteworthy that the fungal strain CF2 tolerated and degraded high concentrations of allethrin up to 1000 mg·L^−1^, which is rarely seen in other microbial strains. In most cases reported to date, the pyrethroid-degrading strains are often inhibited by high concentrations of substrates [65,66,67,68]. Previous studies indicated that the metabolic activity of pyrethroid-degrading microorganisms resulted in complete catabolite repression at high substrate concentrations <200 mg·L^−1^ [18,66,69]. In contrast, high concentration of allethrin (1000 mg·L^−1^) did not inhibit the cell growth of strain CF2, and more importantly the enhanced biodegradation was observed even at the high concentration of allethrin. Moreover, kinetics analysis showed that allethrin half-life was substantially reduced by 507.1 h, as compared to the noninoculated control. The degradation rate constant (0.0193 h^−1^) was much greater than those by other pyrethroid-degrading strains [15,34,43,48]. These findings suggested *F. proliferatum* CF2 has an exceptional ability to degrade allethrin in different ecological niches, which makes it a potent strain for various applications.

## 5. Conclusions

A novel isolated fungal strain, *F. proliferatum* CF2, has the ability to degrade allethrin rapidly without a lag period of growth. This is the first report of biodegradation of allethrin using *F. proliferatum*. The culture conditions of strain CF2 were optimized, and it was found that strain CF2 effectively degraded allethrin over broad pH and temperature ranges. These characteristics indicate that strain CF2 has the potential to bioremediate allethrin-contaminated environments. Furthermore, strain CF2 revealed a remarkable capacity to obtain nutrients by metabolizing allethrin without an extra carbon source. Kinetics analysis showed that the *t*_1/2_ of allethrin with strain CF2 substantially decreased in MSM, from 533.2 to 26.1 hours. These results highlight the promising potential of *F. proliferatum* CF2 in the bioremediation of allethrin-contaminated sites. Furthermore, in enzymatic- and metabolic-based work, it could be more advantageous to understand the detailed mechanism of allethrin degradation with the CF2 strain.

## Figures and Tables

**Table 1:** Central composite design (CCD) for the optimization of allethrin degradation using strain CF2.

Run	*X*_1_	*X*_2_	*X*_3_	Allethrin Degradation (%)
1	+1	+1	−1	76.0 ± 0.88 ^d^
2	0	0	0	93.0 ± 0.57 ^g^
3	0	0	−1.68	75.0 ± 1.10 ^d^
4	0	0	0	93.0 ± 1.70 ^g^
5	+1	−1	−1	69.0 ± 0.57 ^b^
6	−1	−1	−1	64.0 ± 0.57 ^a^
7	0	0	0	94.5 ± 0.28 ^ghi^
8	0	0	0	95.5 ± 0.28 ^hi^
9	−1	+1	−1	73.0 ± 0.00 ^c^
10	0	0	0	95.6 ± 0.57 ^i^
11	−1.68	0	0	76.0 ± 0.57 ^d^
12	0	0	0	93.5 ± 0.57 ^gh^
13	0	0	0	93.0 ± 0.57 ^g^
14	0	+1.68	0	75.0 ± 0.57 ^d^
15	−1	+1	+1	79.0 ± 0.57 ^e^
16	0	0	0	94.3 ± 0.03 ^ghi^
17	−1	−1	+1	85.0 ± 0.00 ^f^
18	0	0	+1.68	85.2 ± 0.11 ^f^
19	+1	+1	+1	71.0 ± 0.60 ^c^
20	0	−1.68	0	75.0 ± 0.00 ^d^
21	+1	−1	+1	85.0 ± 0.00 ^f^
22	0	0	0	95.3 ± 0.57 ^hi^
23	+1.68	0	0	75.0 ± 0.57 ^d^
24	0	0	0	94.5 ± 0.28 ^ghi^

Note: Data presented are the means of three replicates with standard error. Different letters indicate significant differences (*p* < 0.005, least significant (LSD) test) according to the Duncan test.

## Supplementary Materials

Supplementary materials can be found at https://www.mdpi.com/2076-2607/8/4/593/s1.
