# Supplementary material for: Biodegradation of Allethrin by a Novel Fungus Fusarium proliferatum Strain CF2, Isolated from Contaminated Soils
Source: Microorganisms. 2020 Apr 20;8(4):593. doi: 10.3390/microorganisms8040593 (PMC7232317; doi:10.3390/microorganisms8040593)
Supplement: Supplementary file 1 [file microorganisms-08-00593-s001.pdf]

Supplementary information

Biodegradation of allethrin by a novel fungus *Fusarium proliferatum* strain CF2, isolated from contaminated soils

Pankaj Bhatt[1,2], Wenping Zhang[1,2], Ziqiu Lin[1,2], Shimei Pang[1,2], Yaohua Huang[1,2], and Shaohua Chen[1,2*]

[1]*State Key Laboratory for Conservation and Utilization of Subtropical Agro-bioresources, Guangdong Province Key Laboratory of Microbial Signals and Disease Control, Integrative Microbiology Research Centre, South China Agricultural University, Guangzhou 510642, China*;

[2]*Guangdong Laboratory for Lingnan Modern Agriculture, Guangzhou 510642, China*

[*]**Corresponding author**

Shaohua Chen

South China Agricultural University, Guangzhou 510642, China

Tel: +86-20-8528 8229

Fax: +86-20-8528 0292

E-mail: shchen@scau.edu.cn

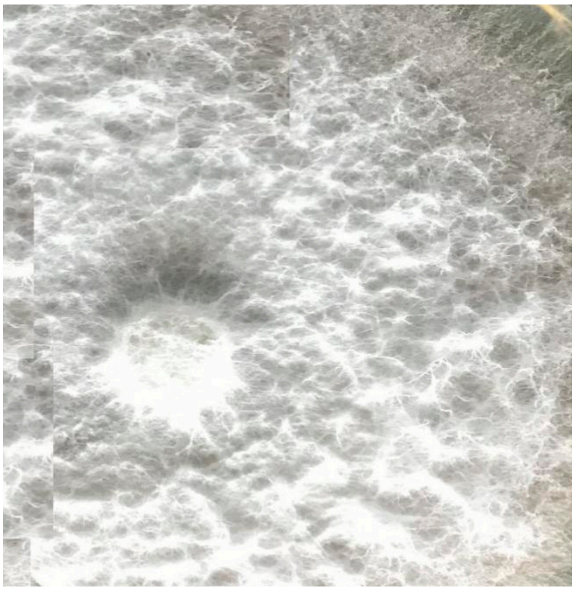

Figure S1 Growth of strain CF2 on Czapek-Dox agar medium

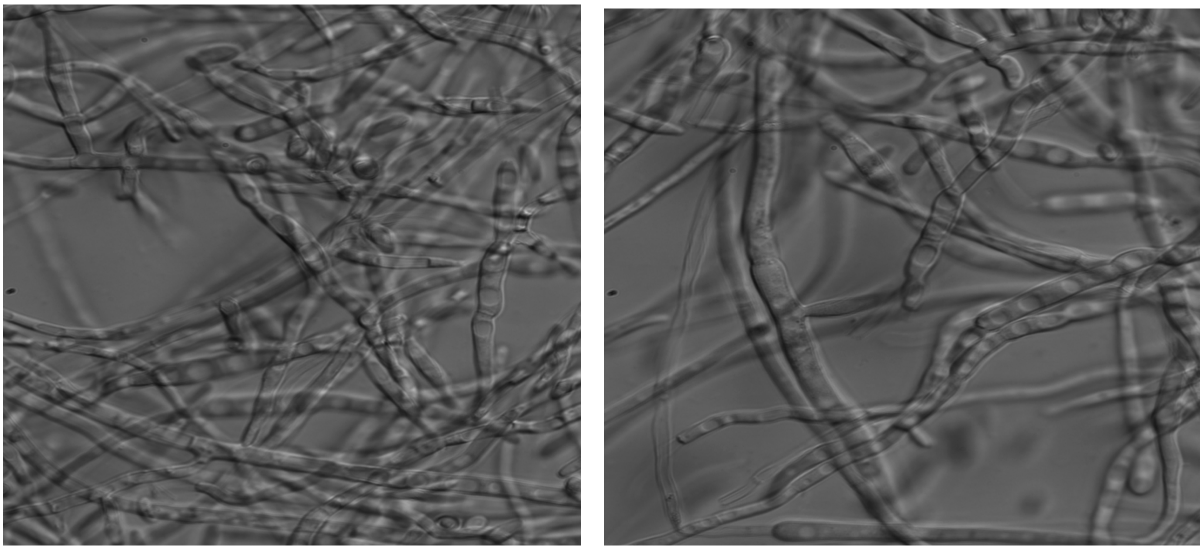

Figure S2 The morphological examination of the strain CF2 using compound microscope (Zeiss LSM 880, Germany)

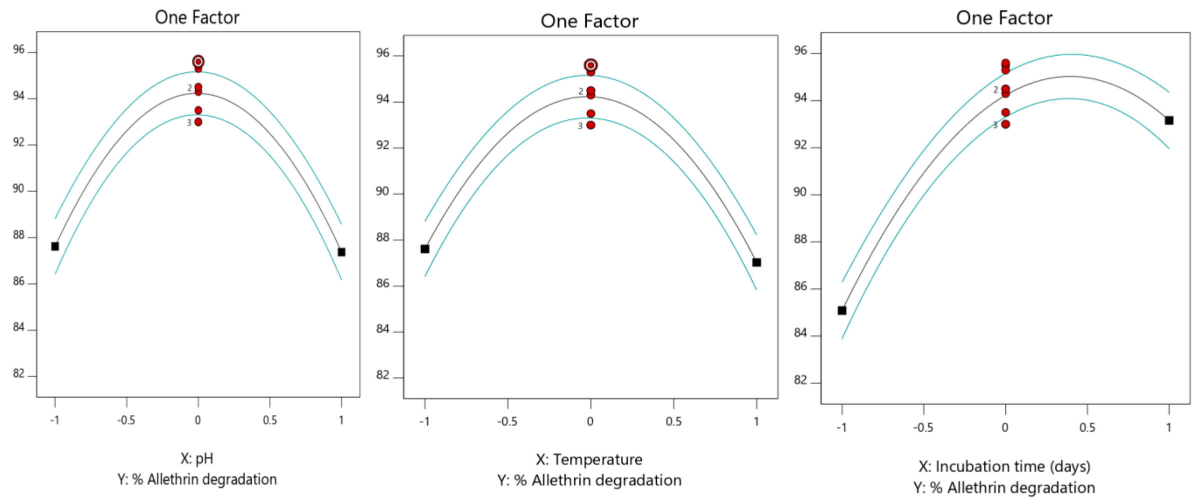

Figure S3 Effect of each of the three parameters (pH, temperature and incubation time) separately on the allethrin degradation

Table S1. Analysis of variance (ANOVA) for the quadratic model in allethrin degradation with strain CF2.

**Source.**	SS	DF	MS	*F*-value	*p*-value
Model	2418.83	9	268.76	143.23	<0.0001
A-A	0.2071	1	0.2071	0.1104	0.7446
B-B	1.17	1	1.17	0.6244	0.4426
C-C	222.74	1	222.74	118.71	<0.0001
AB	12.50	1	12.50	6.66	0.0218
AC	32.00	1	32.00	17.05	0.0010
BC	162.00	1	162.00	86.34	<0.0001
A^2	735.86	1	735.86	392.18	<0.0001
B^2	775.00	1	775.00	413.03	<0.0001
C^2	423.39	1	423.39	225.64	<0.0001
Residual	26.27	14	1.88		
Lack of Fit	16.41	5	3.28	3.00	0.0726
Pure Error	9.86	9	1.10		
Cor Total	2445.10	23			

Note: R^2 = 0.9823, DF refers to the degrees of freedom; SS refers to the sum of squares; MS refers to the mean square. A *p*-value <0.05 indicates that the model terms are significant.
